# Association of Maternal Factors with Perinatal Complications in Pregnancies Complicated with Diabetes: A Single-Center Retrospective Analysis

**DOI:** 10.3390/jcm7010005

**Published:** 2018-01-02

**Authors:** Sho Endo, Yoshifumi Saisho, Kei Miyakoshi, Daigo Ochiai, Tadashi Matsumoto, Yoshinaga Kawano, Masanori Mitsuishi, Junichiro Irie, Masami Tanaka, Shu Meguro, Mamoru Tanaka, Hiroshi Itoh

**Affiliations:** 1 Department of Internal Medicine, Keio University School of Medicine, Tokyo 160-8582, Japan; shoendo0120@yahoo.co.jp (S.E.); kitasato86@z5.keio.jp (Y.K.); mitsumasa@clock.ocn.ne.jp (M.M.); j-irie@z8.keio.jp (J.I.); tana176k@sepia.ocn.ne.jp (M.T.); shumeg@z8.keio.jp (S.M.); hiito@keio.jp (H.I.); 2 Department of Obstetrics and Gynecology, Keio University School of Medicine, Tokyo 160-8582, Japan; kei.z7@keio.jp (K.M.); ochiaidaigo@gmail.com (D.O.); tmatsumoto@a2.keio.jp (T.M.); mtanaka@keio.jp (M.T.)

**Keywords:** pregnancy, type 1 diabetes, type 2 diabetes, perinatal complication

## Abstract

Objective: The aim of this study was to clarify the association of maternal factors with perinatal complications in pregnancies complicated with type 1 (T1D) or type 2 diabetes (T2D). Methods: We conducted a retrospective chart review and enrolled 26 Japanese pregnant women with diabetes who received perinatal care at our hospital between 2008 and 2015. Perinatal complications were defined as one or more of the following: miscarriage, fetal death, fetal dysfunction, fetal structural anomaly, small-for-gestational age, large-for-gestational age (LGA), premature birth, neonatal hypoglycemia, pregnancy-induced hypertension (PIH), deterioration of maternal kidney function, and urgent Caesarean section (CS). The associations between perinatal complications and maternal factors were examined. Results: Approximately 70% and 50% of women with T1D and T2D experienced perinatal complications, respectively. LGA, neonatal hypoglycemia, and urgent CS were major perinatal complications in women with T1D, while PIH and urgent CS were major complications in those with T2D. In women with T1D, pre-gestational HbA1c was significantly higher in women with perinatal complications than in those without. In women with T2D, pre-gestational body mass index was significantly higher in women with perinatal complications than in those without. Conclusions: These findings suggest that while pre-gestational glycemic control remains the most important issue in women with T1D, pre-gestational weight control in addition to glycemic control should be greater emphasized in women with T2D to reduce the risk of perinatal complications.

## 1. Introduction

Maternal blood glucose control during pregnancy in women with diabetes is extremely important for reducing perinatal complications in both the mother and infant [1]. Recently, there has been growing concern regarding the increase in perinatal complications associated with conceiving at a later age as well as the increase in patients with type 2 diabetes (T2D) developing on a background of metabolic syndrome [2]. Although current guidelines for the management of pregnancy in diabetics specify the same strategy for type 1 diabetes (T1D) and T2D, recent studies suggest that different maternal factors are associated with perinatal outcomes between women with T1D and T2D [3]. Identifying maternal factors influencing perinatal complications is important in order to recognize high-risk patients and manage them to improve perinatal outcomes; however, this information remains scarce, especially in the Japanese population.

Therefore, in this study, we examined the maternal factors influencing perinatal complications in pregnant women with T1D and T2D who received childbirth management at our hospital between 2008 and 2015.

## 2. Subjects and Methods

We retrospectively conducted a chart review and identified a total of 26 cases of diabetes with a singleton pregnancy (T1D; 15 cases, T2D; 11 cases) that received perinatal care at our hospital between 2008 and 2015. We excluded one case of twins and one case of steroid-induced diabetes. This study was conducted under the approval of the Ethical Review Board of our hospital (approval number: 20160247, approval date: 31 October 2016). Twenty-five patients had been diagnosed with diabetes and treated prior to pregnancy, while one patient was diagnosed with T2D for the first time during pregnancy.

Information, including the maternal weight before and during pregnancy, body mass index (BMI), HbA1c, glycoalbumin (GA), serum creatinine (Cr), and insulin dose, was extracted from their medical records. A patient was deemed to have perinatal complications when she had one or more of the following conditions; miscarriage, fetal death, fetal dysfunction, fetal structural anomaly, small-for-gestational age (SGA), large-for-gestational age (LGA), preterm birth <34 weeks of gestation, neonatal hypoglycemia, pregnancy-induced hypertension (PIH), worsening of maternal renal function, and urgent Caesarean section (CS). SGA and LGA were defined as values less than the 10th percentile or more than the 90th percentile of birth weight, respectively, depending on the gestational age according to the Japan Pediatric Society [4]. PIH was defined as preeclampsia, gestational hypertension, superimposed preeclampsia, or eclampsia according to the Japan Society of Obstetrics and Gynecology [5]. Worsening of maternal renal function was defined as a more than two-fold increase in serum Cr level during pregnancy. Mean HbA1c during pregnancy was calculated as the average of the values at the following four points: before pregnancy, early pregnancy (8–12 weeks), mid-pregnancy (18–22 weeks), and late pregnancy (28–32 weeks). The number of weeks of pregnancy was calculated based on the last menstrual period, date of ovulation, and crown-rump length using ultrasonography.

All women were treated with insulin in addition to nutritional therapy during pregnancy according to the guidelines of the Japan Diabetes Society [6]. In women with T1D, 12 patients received basal-bolus therapy and three received continuous subcutaneous insulin infusion, while in women with T2D, all patients received basal-bolus therapy. The patients were instructed to conduct self-monitoring of blood glucose (SMBG) seven times a day: before and 2 h after each meal and at bedtime. Glycemic targets were 70 mg/dL to 100 mg/dL for fasting plasma glucose and <120 mg/dL for 2 h after meals, and insulin dosage was adjusted based on the SMBG values.

Regarding the presence of perinatal complications and their relationship with maternal factors, comparisons between the two groups were made using *t*-test or chi-squared test. Multivariate regression analysis was performed using the Statistical Package for the Social Sciences (IBM SPSS Statistics version 23, Armonk, NY, USA). Data are presented as mean ± standard deviation (SD), and values with *p* < 0.05 were considered statistically significant.

## 3. Results

Table 1 shows the patient background characteristics. Age at pregnancy was significantly younger (34 ± 6 vs. 38 ± 3 years, *p* = 0.04) and duration of diabetes was significantly longer in women with T1D than in those with T2D (17.7 ± 7.9 vs. 3.6 ± 4.2 years, *p* < 0.0001). Pre-gestational body weight and BMI were significantly lower in women with T1D than in those with T2D (body weight 54.2 ± 7.6 vs. 69.6 ± 13.8 kg, *p* < 0.01, BMI 21.0 ± 2.4 vs. 27.5 ± 4.9 kg/m^2^, *p* = 0.0001). While none of the women with T1D were obese (i.e., BMI ≥ 25), obesity was observed in 6 of 11 women with T2D (0 vs. 54.5%, *p* = 0.001). Weight gain during pregnancy was significantly greater in women with T1D than in those with T2D (11.6 ± 5.1 vs. 5.8 ± 4.9 kg, *p* = 0.008). While the amount of insulin used before pregnancy was significantly higher in women with T1D (38.9 ± 15.3 vs. 9.5 ± 20.0 U/day, *p* = 0.0004), the increment in insulin dosage during pregnancy was significantly greater in women with T2D (5.4 ± 17.4 vs. 35.4 ± 41.7 U/day, *p* = 0.02). Pre-gestational HbA1c tended to be higher in women with T1D, but the difference was not statistically significant (7.6 ± 1.8% vs. 7.1 ± 1.6%, *p* = 0.52). There was no significant difference in the proportion of women who developed perinatal complications between those with T1D and T2D (67% vs. 45%, *p* = 0.28).

In women with T1D, perinatal complications were observed in 11 of 15 cases (Table 2). The majority were LGA, neonatal hypoglycemia, and urgent CS. Two cases with PIH underwent urgent CS because of poor blood pressure control at 32 and 34 weeks of gestation, respectively. Regarding the two patients with worsening maternal renal function, their serum Cr level increased from 1.3 mg/dL to 2.7 mg/dL and from 0.9 mg/dL to 2.0 mg/dL, respectively; the former case underwent urgent CS because of poor blood pressure control at 34 weeks of gestation. One case with fetal dysfunction (non-reassuring fetal heart rate monitoring) underwent urgent CS.

Table 3 shows the relationship between perinatal complications and maternal factors in women with T1D. In women with perinatal complications, pre-gestational HbA1c and mean HbA1c during pregnancy were significantly higher than in women without complications (pre-gestational HbA1c 8.2 ± 1.7% vs. 6.0 ± 0.3%, *p* = 0.03, mean HbA1c 7.2 ± 0.9% vs. 6.0 ± 0.6%, *p* = 0.04). In women with T1D, perinatal complications were observed in eight cases with pre-gestational HbA1c ≥ 7% (*n* = 9) and in two cases with pre-gestational HbA1c < 7% (*n* = 6, 89% vs. 33%, *p* = 0.03). Five of the six LGA cases and four of the five neonatal hypoglycemia cases developed in the group with pre-gestational HbA1c ≥ 7%. The association between pre-gestational HbA1c and perinatal complications remained significant after adjustment for age and pre-gestational BMI in multivariate regression analysis (β = 0.64, *p* = 0.02).

In women with T2D, perinatal complications were observed in six of 11 cases, in which the majority were PIH and urgent CS (Table 2). Three of the four PIH patients had had hypertension prior to pregnancy. Two of the six PIH cases underwent urgent CS. One case had fetal structural anomaly (congenital heart disease). Table 4 shows the relationship between perinatal complications and maternal factors in the T2D group. In women with complications, pre-gestational BMI was significantly higher than in those without complications (31.1 ± 3.3 vs. 23.2 ± 1.0 kg/m^2^, *p* = 0.001). While no perinatal complications developed in any of the five patients who were not obese, perinatal complications developed in all patients who were obese (100% vs. 0%, *p* = 0.001). Multivariate regression analysis including age, pre-gestational BMI, and pre-gestational HbA1c also revealed that age and BMI but not HbA1c were associated with perinatal complications (β = 0.47, 0.78, and −0.01, *p* = 0.004, <0.001, and 0.94, respectively).

## 4. Discussion

In this study, we found that the incidence of perinatal complications was associated with pre-gestational HbA1c in women with T1D, whereas it was associated with pre-gestational BMI in women with T2D. The findings from the present study suggest that the maternal factors influencing perinatal complications may differ between pregnant women complicated with T1D and T2D.

The differences in patient backgrounds between women with T1D and T2D were consistent with a previous report in Japan [7]. Duration of diabetes was longer and incidence of retinopathy was higher in women with T1D, while pre-gestational BMI and incidence of obesity were higher in those with T2D. It was noted that pre-gestational HbA1c level was not significantly different between women with T1D and T2D, most of whom had been receiving pre-gestational care for diabetes.

In this study, perinatal complications were observed in approximately 70% of women with T1D, in which the majority were the development of LGA, neonatal hypoglycemia, and urgent CS. Pre-gestational HbA1c was associated with a risk of perinatal complications, and indeed, there was a significantly higher incidence of perinatal complications in women with pre-gestational HbA1c ≥ 7% than in those with pre-gestational HbA1c < 7%. Furthermore, in women with complications, mean HbA1c during pregnancy as well as HbA1c and GA at delivery were also higher than those in women without complications, suggesting that sustained hyperglycemia during pregnancy likely influenced the development of LGA and neonatal hypoglycemia. These findings suggest that pre-pregnancy care including glycemic control and planning of conception remains most important to reduce perinatal complications in women with T1D.

Perinatal complications were observed in approximately 50% of women with T2D, of which the majority were the development of PIH and urgent CS. In contrast to women with T1D, pre-gestational BMI was associated with a risk of perinatal complications, and indeed, all women who developed perinatal complications were obese. Maternal obesity has been shown to associate with perinatal complications such as PIH and CS [8]. Maternal obesity has been shown to be an independent risk factor for PIH, even in pregnant women without gestational diabetes or impaired glucose tolerance [9,10]. Microangiopathy, advanced age, and hypertension have also been reported to be risk factors for PIH [11,12]. In this study, in addition to maternal obesity, these factors might also have increased the incidence of PIH. On the other hand, while obesity is considered a risk factor for LGA, the incidence of LGA was low in this study compared with a previous study [13]. This may be because the patients in this study adhered well to their nutritional management during pregnancy, and achieved optimal weight gain during pregnancy.

In this study, it was noted that pre-gestational HbA1c was not associated with perinatal complications in women with T2D. It is well appreciated that maternal glycemic control reduces perinatal complications in women with diabetes, and current guidelines recommend a similar treatment strategy for both women with T1D and T2D [14]. However, it remains unclear whether the same treatment strategy for both T1D and T2D is optimal. A recent meta-analysis has shown that despite less severe glycemic disturbance, women with T2D had no better perinatal outcomes than those with T1D, suggesting that factors other than glycemic control also affect perinatal complications in women with T2D [15]. In the present study, there was no significant difference in pre-gestational HbA1c and mean HbA1c during pregnancy between T2D women with and without perinatal complications. Also, the incidence of perinatal complications was not significantly different between T2D women with pre-gestational HbA1c ≥ 7% compared with those with pre-gestational HbA1c < 7% (50% vs. 43%, *p* = 0.82).

Taken together, the above findings regarding pregnancy in T2D on a background of metabolic syndrome suggest that not only glycemic control but also obesity before pregnancy may greatly influence the risk of perinatal complications. Owens et al. reported that types of perinatal complications differ between women with T1D and T2D, and despite the fact that HbA1c before pregnancy in women with T2D is lower than in those with T1D, women with T2D still have an excess risk of perinatal complications, suggesting that factors other than the blood glucose level play an important role [3]. Handisurya et al. also reported that BMI and blood glucose control influence perinatal complications [16]. The present findings were in line with these previous reports, suggesting that weight control before pregnancy is important for obese patients with T2D in order to reduce the risk of perinatal complications. Omori et al. reported that perinatal outcome was worse in Japanese women with T2D compared with those with T1D in 1994 [17], while a more recent study in Japan showed comparable perinatal outcomes between women with T1D and T2D [7], suggesting an improvement of perinatal outcome in women with T2D possibly due to improvement of the pre-conception management of T2D. Recently, advances in insulin therapy have also facilitated optimal glycemic control during pregnancy in women with T2D. This indicates that control of weight as a residual risk factor may become an issue to address in the future.

The present study has limitations. First, our study had a retrospective cross-sectional design. Thus, we are not able to exclude the possibility of the presence of residual confounders. Also, the causal relationship remains uncertain. Second, the sample size was small. Thus, we were not able to conduct analyses for each complication because of the limited number of subjects for each complication. Also, due to the insufficient statistical power, we may not be able to detect other factors related to perinatal complications. On the other hand, some of the findings might be a chance finding because of the multiple comparisons; however, the main findings of this study showed high statistical significance and we believe that our conclusion is robust. Because of these limitations, the results of the present study should be carefully interpreted. Nonetheless, we believe that our findings have an important implication for the clinical management and treatment of pregnant women with diabetes. To confirm our findings, future studies with a prospective design and larger sample size including women with and without perinatal complications are warranted.

## 5. Conclusions

Poor pre-gestational glycemic control and obesity were strongly associated with perinatal complications in women with T1D and T2D, respectively. While pre-gestational glycemic control remains the most important issue in women with T1D, the importance of not only pre-gestational glycemic control but also weight control should be emphasized in women with T2D to improve their perinatal outcomes.

## Figures and Tables

**Table 1:** Patient characteristics.

	All	Women with T1D	Women with T2D	*p*
*n* (%)	26 (100)	15 (58)	11 (42)	
Age (years)	35.7 ± 5.1	34.0 ± 5.8	38.0 ± 2.7	0.04
Disease duration (years)	11.7 ± 9.6	17.7 ± 7.9	3.6 ± 4.2	<0.01
Nullipara, *n* (%)	19 (73.1)	12 (80.0)	7 (63.6)	0.35
Pre-gestational body weight (kg)	60.8 ± 4.7	54.2 ± 7.6	69.6 ± 13.8	<0.01
Pre-gestational BMI (kg/m^2^)	23.8 ± 4.8	21.0 ± 2.4	27.5 ± 4.9	<0.01
Obesity, *n* (%)	6 (23.1)	0 (0)	6 (54.5)	<0.01
Hypertension, *n* (%)	4 (15.4)	1 (6.7)	3 (27.2)	0.15
Diabetic retinopathy, *n* (%)	10 (38.5)	7 (46.7)	3 (27.2)	0.32
Pre-gestational serum creatinine (mg/dL)	0.69 ± 0.39	0.66 ± 0.2	0.74 ± 0.6	0.61
Chronic kidney disease (eGFR < 60 mL/min), *n* (%)	2 (7.7)	1 (6.7)	1 (9.1)	0.82
Pre-gestational HbA1c (%)	7.4 ± 1.7	7.6 ± 1.8	7.1 ± 1.6	0.52
Insulin dose before pregnancy (U/day)	26.0 ± 22.7	38.9 ± 15.3	9.5 ± 20.0	<0.01
Weight gain during pregnancy (kg)	9.1 ± 5.7	11.6 ± 5.1	5.8 ± 4.9	<0.01
Increment in insulin during pregnancy (U/day)	18.6 ± 33.5	5.4 ± 17.4	35.4 ± 41.7	0.02

*p*, Women with T1D vs. those with T2D; T1D, type 1 diabetes; T2D, type 2 diabetes; BMI, body mass index; eGFR, estimated glomerular filtration rate.

**Table 2:** Perinatal complications.

Women with T1D (*n* = 15)	Women with T2D (*n* = 11)
LGA (6)	PIH (4)
Neonatal hypoglycemia (5)	Urgent Caesarean section (3)
Urgent Caesarean section (5)	Neonatal hypoglycemia (2)
PIH (2)	SGA (1)
Worsening maternal renal function (2)	LGA (1)
Fetal dysfunction (1)	Fetal structural anomaly (1)
Preterm birth <34 weeks of gestation (1)	

Number of cases is given in parentheses. LGA, large-for-gestational age; PIH, pregnancy-induced hypertension; SGA, small-for-gestational age.

**Table 3:** Relationship between perinatal complications and maternal factors in women with T1D.

	Perinatal Complications (+)	Perinatal Complications (−)	*p*
*n* (%)	11 (73)	4 (27)	
Age (years)	35.2 ± 4.9	30.8 ± 6.5	0.20
Disease duration (years)	19.5 ± 7.1	12.5 ± 7.4	0.13
Pre-gestational BMI (kg/m^2^)	21.3 ± 2.1	20.4 ± 2.8	0.54
Pre-gestational serum creatinine (mg/dL)	0.70 ± 0.2	0.60 ± 0.1	0.53
Pre-gestational HbA1c (%)	8.2 ± 1.7	6.0 ± 0.3	0.03
HbA1c at delivery (%)	6.7 ± 0.8	6.0 ± 0.6	0.13
GA at delivery (%)	17.3 ± 2.2	16.5 ± 2.1	0.57
Mean HbA1c during pregnancy (%)	7.2 ± 0.9	6.0 ± 0.6	0.04
Weight gain during pregnancy (kg)	12.5 ± 5.6	9.1 ± 2.3	0.27
Increment in insulin during pregnancy (U/day)	2.7 ± 18	12.3 ± 13	0.38

*p*, Women with perinatal complications vs. those without perinatal complications; BMI, body mass index; GA, glycoalbumin.

**Table 4:** Relationship between perinatal complications and maternal factors in women with T2D.

	Perinatal Complications (+)	Perinatal Complications (−)	*p*
*n* (%)	6 (55)	5 (45)	
Age (years)	39.3 ± 2.5	36.2 ± 1.9	0.07
Disease duration (years)	5.2 ± 4.6	1.8 ± 1.6	0.19
Pre-gestational BMI (kg/m^2^)	31.1 ± 3.3	23.2 ± 1.0	0.001
Pre-gestational serum creatinine (mg/dL)	0.8 ± 0.7	0.7 ± 0.1	0.65
Pre-gestational HbA1c (%)	7.0 ± 1.0	7.2 ± 2.0	0.89
HbA1c at delivery (%)	5.9 ± 0.2	6.2 ± 1.2	0.55
GA at delivery (%)	13.3 ± 1.7	14.5 ± 2.6	0.42
Mean HbA1c during pregnancy (%)	6.0 ± 0.5	6.4 ± 1.7	0.67
Weight gain during pregnancy (kg)	4.3 ± 2.7	7.7 ± 5.8	0.27
Increment in insulin during pregnancy (U/day)	31.7 ± 42	39.8 ± 36	0.77

*p*, Women with perinatal complications vs. those without perinatal complications; BMI, body mass index; GA, glycoalbumin.
